# Describing antimicrobial use and reported treatment efficacy in Ontario swine using the Ontario swine veterinary-based Surveillance program

**DOI:** 10.1186/1746-6148-9-238

**Published:** 2013-12-01

**Authors:** Shiona K Glass-Kaastra, David L Pearl, Richard J Reid-Smith, Beverly McEwen, Scott A McEwen, Rocio Amezcua, Robert M Friendship

**Affiliations:** 1 Department of Population Medicine, University of Guelph, 50 Stone Road East, Guelph, Ontario N1G2W1, Canada; 2 Laboratory for Foodborne Zoonoses, Public Health Agency of Canada, 160 Research Lane, Guelph, Ontario N1G B52, Canada; 3 Department of Pathobiology, University of Guelph, 50 Stone Road East, Guelph, Ontario N1G2W1, Canada; 4 Animal Health Laboratory, University of Guelph, 419 Gordon Street, Guelph, Ontario N1G2W1, Canada

**Keywords:** Treatment failure, Treatment efficacy, Antimicrobial use, Swine

## Abstract

**Background:**

The objective of this work was to retrospectively assess records received through the Ontario Swine Veterinary-based Surveillance program July 2007 – July 2009 to describe and assess relationships between reported treatment failure, antimicrobial use, diagnosis and body system affected.

**Results:**

Antimicrobial use occurred in 676 records, 80.4% of all records recording treatment (840). The most commonly used antimicrobials were penicillin (34.9%), tetracyclines (10.7%) and ceftiofur (7.8%), and the use of multiple antimicrobials occurred in 141/676 records (20.9%). A multi-level logistic regression model was built to describe the probability of reported treatment failure. The odds of reported treatment failure were significantly reduced if the record indicated that the gastro-intestinal (GI) system was affected, as compared to all other body systems (p < 0.05). In contrast, the odds of reported treatment failure increased by 1.98 times if two antimicrobials were used as compared to one antimicrobial (p = 0.009) and by 6.52 times if three or more antimicrobials were used as compared to one antimicrobial (p = 0.005). No significant increase in reported treatment failure was seen between the use of two antimicrobials and three or more antimicrobials. No other antimicrobials were significantly associated with reported treatment failure after controlling for body system and the number of antimicrobials used.

**Conclusions:**

Failure of antimicrobial treatment is more likely to occur in non-GI conditions, as compared to GI conditions and the use of multiple antimicrobial products is also associated with an increased probability of antimicrobial treatment failure. The authors suggest that a more preventative approach to herd health should be taken in order to reduce antimicrobial inputs on-farm, including improved immunity via vaccination, management and biosecurity strategies. Furthermore, improved immunity may be viewed as a form of antimicrobial stewardship to the industry by reducing required antimicrobial inputs and consequently, reduced selection pressure for AMR.

## Background

Antimicrobial resistance (AMR) threatens the efficacy of antimicrobial drugs for treating infections in humans and animals alike. Antimicrobial resistance emerges in the presence of antimicrobial products and the transfer of resistance genes among bacteria may occur [1]. As clinical infection with resistant pathogens may lead to prolonged morbidity, increased costs and increased risk of mortality, AMR is a serious concern for food-animal production. Recently, it has been reported that multiple-class resistance within clinical pathogens of swine in Ontario is relatively common [2]. However, it is not known if this has led to increased antimicrobial treatment as there is currently no accurate measurement of the volume of antimicrobial use in Ontario swine. Furthermore, these resistance data do not include information regarding the treatment provided, or efficacy of the treatment. Given that in-vitro resistance does not necessarily predict failure of antimicrobial treatment in-vivo [3], industry stakeholders may also benefit from the knowledge of significant predictors for reporting treatment failure.

In July 2007, the Ontario Swine Veterinary-based Surveillance (OSVS) program was initiated to assess the potential for developing a practitioner-based syndromic health surveillance system for swine in Ontario [4]. Recruited practitioners were asked to record and report on all farm visits or calls related to swine. These reports requested information regarding suspected diagnosis, the body system affected, treatment(s) provided, and the efficacy of treatments. Consequently, these records may provide a valuable source of antimicrobial use and in-vivo efficacy data for Ontario; a complement to the available in-vitro resistance data from the Animal Health Laboratory [5]. Furthermore, these data allow for the examination of associations between treatment with certain antimicrobials and reported treatment failure. Therefore, the objective of this work was to retrospectively assess records received through the OSVS program from July 1, 2007 through June 30, 2009 to describe and assess relationships between reported treatment failure, antimicrobial use, diagnosis, and body system affected.

## Methods

A full description of the OSVS pilot project is available elsewhere [4]. Briefly, the OSVS pilot program was funded by the Ontario Ministry of Agriculture Food & Rural Affairs (OMAFRA) and the Ontario Animal Health Strategic Investment (AHSI) fund. During the July 1 2007 to June 30 2009 time period, reports were received from up to ten practitioners representing five clinics known to service most of the swine industry in Ontario. During this period, practitioners recorded data for all daily swine-related farm visits and calls using either a paper or electronic submission via personal digital assistants (PDA) or an internet-based form. Data collected included whether the visit/call was a disease or routine visit, unique practitioner ID, unique farm ID, signs/symptoms displayed, diagnosis, body system affected, whether it was an incident or ongoing condition, farm history of the condition, diagnosis, veterinarian-prescribed treatment, and efficacy of treatment. As records were made at the visit level, a record could reflect treatment and efficacy at the individual, pen, or herd level.

A database was created through the electronic form submissions and manual input of the paper forms, using Microsoft Access (2003). Data cleaning, tabulations and multi-level logistic regression modeling were performed in Stata/MP 12.1 (Stata Corporation, College Station, Texas, USA). Manual mining of free-text fields was performed in order to determine the most commonly used antimicrobials and common diagnoses. Treatment failure was deemed to have occurred if practitioners recorded a treatment as being not efficacious, or “occasionally” efficacious. Due to small numbers of observations, the nervous, integument and reproductive body systems were combined into a single 'other’ category. The treatment variable was searched to create binary variables for each antimicrobial used and a variable describing the number of antimicrobials used was developed by adding across these binary variables. Therefore, multiple antimicrobial treatment was defined as any record with >1 antimicrobial listed within the treatment text field. Multiple antimicrobial treatments may not have been initiated at the time of the record, but were either 1) in use concurrently, or 2) recently used to treat/control the specific condition in the animals being seen at the time of the visit. As only a single record was found with more than 3 antimicrobials used, a “3 or more” category was created in the number of antimicrobials variable.

Multilevel logistic regression models were built to describe the probability of treatment failure, given antimicrobial treatment. Two- and three-level models were built using practitioner and farm as random intercepts, and the inclusion of a random slope for practitioner was also tested. Fixed effect predictors examined were diagnosis, body system affected, the number of antimicrobials used and each of the individual antimicrobial use variables. A manual backwards-selection process was used to build the model; all predictors were added to the model initially and removed one at a time based on the highest p-value. Categorical variables with > 2 levels were assessed for significance using the likelihood ratio test [6]. As predictors were removed, their impact on all other statistically significant coefficients was assessed to ensure that confounding variables remained in the model; a 30% change in any significant coefficient resulted in the removed variable being replaced in the model [6]. All two-way interaction terms were generated between all significant main-effects and tested within the model at p < 0.05. Where more than one model fit the data, the model with the most negative Akaike information criteria was chosen [6]. At the record level, Pearson and deviance residuals were visualized and any covariate patterns showing anomalous values were recorded. The normality of the best linear unbiased predictors (BLUPS) was assessed visually with normal quantile plots. Models were re-run with the exclusion of noted covariate patterns to assess any dramatic changes in the coefficients. Contrast statements were used to make comparisons between dummy variable categories within the body system and number of antimicrobials variables and the latent variable technique was employed to calculate variance components at each level [7].

## Results

In total, 3691 records were received by the OSVS program from July 2007 – July 2009. Antimicrobial use was reported in 676 of these records. These reflected reports from nine practitioners, submitting 7 to 255 records each and reflected data from 335 farms with 1 to 14 records each. A number of records indicated that an antimicrobial was used, without naming the product (182/676). When drug names were included, the most commonly used antimicrobials were penicillin (34.9%), tetracyclines (10.7%) and ceftiofur (7.8%) (Table 1). Use of multiple antimicrobials in a single record was not uncommon; 141 (20.6%) records indicated that treatment included ≥ 2 antimicrobials (Table 2). The most common combinations of antimicrobials used for treatment were penicillin with tetracyclines (24 records), penicillin with ceftiofur (16 records) and penicillin with a sulfonamide product (13 records) (Table 3).

**Table 1:** Number of OSVS records where treatment with each antimicrobial was reported (July 1, 2007 – June 30, 2009)

**Antimicrobial**	**Frequency**	**Percent of records with antimicrobial treatment (N = 676)**
Penicillin	236	34.91
Specific drug not recorded	182	26.92
Tetracyclines	72	10.65
Ceftiofur	53	7.84
Lincomycin	41	6.07
Neomycin	41	6.07
Tylosin	36	5.33
Sulfonamides	28	4.14
Tulathromycin	26	3.85
Amoxicillin	22	3.25
Florfenicol	20	2.96
Trimethoprim-sulfamethoxazole	19	2.81
Tilmicosin	18	2.66
Gentamicin	14	2.07
Oxytetracycline	11	1.63
Apramycin	10	1.48
Trimethoprim	8	1.18
Spectinomycin	3	0.44
Bacitracin	3	0.44
Tiamulin	3	0.44
Virginiamycin	1	0.15

**Table 2:** Number of OSVS records where treatment efficacy or failure was reported by the number of antimicrobials used for treatment (July 1, 2007 – June 30, 2009)

**Number of antimicrobials used**	**1**	**2**	**3**	**4**	**Total**
Treatment failure	360	86	26	1	473
Efficacious treatment	175	26	2	0	203
Total	535	112	28	1	

**Table 3:** Most frequent antimicrobial pairings as reported within OSVS records (July 1, 2007 – June 30, 2009)

**Combination**	**Number of records (N = 676)**
Penicillin + tetracycline	24
Penicillin + ceftiofur	16
Penicillin + sulfonamide	13
Sulfonamide + tetracycline	9
Neomycin + tetracycline	8
Tulathromycin + ceftiofur	8
Sulfonamide + trimethoprim	8
Penicillin + sulfonamide + tetracycline	7

In records with antimicrobial treatments recorded, the recorded body systems affected were: respiratory, GI, musculoskeletal, multisystemic, or other. More than 27% (185/676) of records with antimicrobial use indicated that multiple systems were affected. Furthermore, 78.9% (146/185) of these records indicated treatment failure. The second and third most commonly noted body systems affected were respiratory and GI. Treatment failure was reported in 74.3 and 52.9% of these records, respectively. The most commonly noted diagnoses were *Streptococcus suis* infection (94 records, 13.9%) and porcine reproductive and respiratory syndrome (PRRS) (93; 13.8%) (Table 4). Interestingly, antimicrobials were used in records where diagnoses included non-bacterial conditions (e.g. porcine circovirus infection and influenza) (Table 4).

**Table 4:** Diagnoses recorded in OSVS records that indicated antimicrobial use, when antimicrobials were used in treatment (July 1, 2007 – June 30, 2009)

**Diagnosis**	**Number of records**	**Proportion of records**
*Streptococcus suis*	94	13.91
PRRS	93	13.76
Scours^a^	49	7.25
*Escherichia coli*	45	6.66
Circovirus	43	6.36
Arthritis	40	5.92
Ileitis	31	4.59
Influenza	30	4.44
Erysipelas	29	4.29
Glassers	28	4.14
Greasy pig^a^	19	2.81
*Actinobacillus pleuropneumonia*	19	2.81
*Salmonella*	18	2.66
Lameness	17	2.51
Mycoplasma	17	2.51
Coccidiosis	10	1.48

^a^Specific pathogen not indicated.

Significant predictors within the final multi-level logistic regression model describing treatment failure included body system affected, the number of antimicrobials used and the use of neomycin (Table 5). Practitioners and farm were included as random effects, accounting for the 3-level nested structure of the data (reports from farms within a veterinarian’s practice) (Table 5). No significant interaction terms were found. Records indicating GI disease were at significantly decreased odds of treatment failure as compared to multisystemic, musculoskeletal and respiratory body systems (Table 5). No other significant differences in treatment failure were present between body systems. As the number of antimicrobials used increased, so did the odds of treatment failure. The odds of failure increased by 2.29 times if two antimicrobials were used as compared to one antimicrobial (p <0.01) and by 7.56 times if three or more antimicrobials were used as compared to one antimicrobial (p = 0.01). No significant increase in treatment failure was seen between the use of two antimicrobials and three or more antimicrobials (OR = 1.19; p = 0.15; CI: -0.45 – 2.84). Finally, reduced odds of treatment failure was found in records where neomycin was used (OR = 0.34; p = 0.02). No other antimicrobials were significantly associated with treatment failure after controlling for body system affected.

**Table 5:** Odds ratios and p-values for the mixed logistic regression model describing the effect of body system treated, number of antimicrobials used and the use of neomycin upon treatment failure in 676 OSVS records, when antimicrobials were used in treatment (July 1, 2007 – July 30, 2009)

**Fixed effects**	**Odds ratio**	**Standard error**	**P-value**
System			0.01
GI	Referent	-	-
Multisystemic	3.14	0.99	<0.01
Musculoskeletal	2.39	1.00	0.04
Other	1.96	0.76	0.08
Respiratory	2.40	0.75	<0.01
Not recorded	1.47	0.78	0.46
Number of antimicrobials used			0.01^a^
1	Referent	-	-
2	2.29	0.72	<0.01
3 or more	7.56	6.10	0.01
Neomycin	0.34	0.15	0.02
Intercept	1.02	0.41	0.97
Random intercepts	Variance	Standard error	P-value
Practitioner	0.81	0.62	<0.01^b^
Farm	0.60	0.39	

^a^P-value for the likelihood ratio test comparing the model with and without the system variable.

^b^P-value for the likelihood ratio test comparing the random effects model to the fixed-effects model alone.

The variance partition coefficient indicated that the majority of variation in reported treatment failure occurred at the report level after accounting for fixed effects (70.0%) as compared to the practitioner (17.2%) and farm levels (17.8%).

The BLUPS at the farm and practitioner levels were normally distributed by visual assessment. No anomalous values for the Pearson or deviance residuals were apparent at the report level.

## Discussion and conclusion

This work presents an assessment of the use of antimicrobials in the Ontario swine industry and the frequency of treatment failure when antimicrobials were used for treating disease. Results here support other reports of common antimicrobial use within the swine industry [1,8]. Similarly, the most commonly used antimicrobials in the OSVS dataset were consistent with the three highest-use injectable antimicrobials reported by CIPARS [9]. Although the administration route for antimicrobials is available within the CIPARS data, the reasons for use are not available. However, sentinel practitioner systems such as the OSVS may provide insight on the proportion of antimicrobial use for disease treatment as compared to growth promotion or prophylaxis, as OSVS participants were requested to only record treatments (not routine use). Use of multiple antimicrobials was not uncommon within the reports assessed here; more than 20% of records indicated that ≥ 2 antimicrobials were used for treatment. Furthermore, these data displayed that antimicrobials were used in cases where the expected diagnosis was viral (e.g., porcine circovirus infection and influenza) or non-infectious (e.g., injury), either as a precautionary measure if a suspected viral infection is actually bacterial, or to prevent/treat secondary bacterial infections.

In reports indicating antimicrobial use, treatment failure was surprisingly high (70%). To assess predictors for treatment failure within these records, a multi-level logistic regression model was built with the assumption that the reported treatment failure reflected failure of the antimicrobial mentioned in the treatment field. Results of this model indicated that the odds of treatment failure was associated with the body system affected, the number of antimicrobials used in treatment and use of the antimicrobial neomycin. The odds of treatment failure was significantly lower when the GI system was affected as compared to respiratory, multisystemic, musculoskeletal or other body system conditions, and the odds of treatment failure with multisystemic conditions was significantly higher than in reports with no body system recorded.

Significant differences in treatment failure among body systems affected likely reflected the etiology of common swine conditions. Many respiratory and multi-systemic conditions in swine have a viral etiology (e.g., porcine circovirus infection, influenza, PRRS) and antimicrobial treatment is not expected to resolve the primary viral infection. This is not to say that antimicrobial treatment is always inappropriate however, as secondary bacterial infections or co-infections may also occur and antimicrobial treatment may prevent the exacerbation of clinical signs [10,11]. Accordingly, prudent use guidelines for antimicrobial use in swine encourage veterinarians to determine the causative agent of disease while recognizing the potential for secondary bacterial infections [12].

The differences in the probability of treatment failure among the body systems may also reflect the route of administration of antimicrobials in swine production. Due to large herd or group sizes, treatment of individual animals by injection can be difficult and impractical. Therefore, mass medication of herds through water or feed is a common practice [9]. Enteric bacterial infections are expected to be effectively treated with this method, given that no metabolism or uptake of the drug into the blood stream/tissue is required. As such, failure of orally administered treatment is expected to occur more often than when other body systems are affected. Furthermore, ill animals are likely to go off-feed, which can result in under-dosing of infections requiring drug metabolism or blood stream uptake. The observed sparing effect of neomycin use may also be explained by body system and route of administration; neomycin is supplied through feed or water in order to treat bacterial enteritis caused by *Escherichia coli* and *Salmonella* spp. [13].

The odds of treatment failure increased significantly with the use of multiple antimicrobials. Although practitioners were requested to record data only pertaining to the current visit, the data suggested that records listing multiple antimicrobials for treatment may have reflected either concurrent use of multiple products, or successive use following failure of the primary treatment. However, these results suggest that it may be prudent to explore non-bacterial etiologies and preventative approaches to swine health when the use of two or more antimicrobials is being considered.

Given that the variance in reported treatment failure was greater at the report level than the farm or practitioner level, it may be assumed that the disease in question has greater influence on the probability of treatment failure than farm- or practitioner-level factors. Therefore, the potential impact of prescription-only standards for accessing antimicrobials on-farm is great, as suggested by the Veterinary Drugs Directorate “Uses of antimicrobials in food animals in Canada” report [14]. These standards would require producers to obtain a prescription for all antimicrobial use, which is not a current practice in Ontario. Upon the adoption of this recommendation, a shift in the influence may occur towards practitioners. The impact of this shift upon the frequency of treatment failure presents an interesting topic for follow-up studies.

In instances of non-GI conditions or failure of first-line antimicrobial treatment, a review of the vaccination, biosecurity, artificial insemination, and air quality strategies used on-farm may provide a more effective means of improving and maintaining herd health. Vaccines are available and a topic of ongoing research for many common pathogens of swine, including *S. suis*[15,16], PRRS [17,18], porcine circovirus [19,20], and *E. coli*[21,22]. Given that the majority of antimicrobial use reported in the OSVS reports reflected the treatment of conditions caused by these pathogens, successful vaccination strategies are expected to lower the probability of antimicrobial use and treatment failure alike. Similarly, two manageable biosecurity measures, the presence of a shower on-site and limited access to main entrances by rendering trucks, have been shown to be associated with reduced probability of positive PRRS virus status on-farm [23]. Other management practices such as the use of semen from specific-pathogen free boars for artificial insemination [24], weaning at 28 days of age or later [25] improving ventilation [26,27], reducing group sizes to decrease density [28] and switching to all-in-all-out flow systems [28] have been shown to be associated with reduced probability of positive viral status. However, the practicality of some of these changes is questioned, as many may require large economic inputs by producers (e.g., changes requiring renovations to facilities). Therefore, it may be prudent for practitioners, producers, and policy makers to reassess the current guidelines around vaccinations, and the use and acquisition of antimicrobials in swine production. In order to reduce the volume of antimicrobial product being used to treat non-bacterial infections, priority should be given to research that focuses on assessing the health and economic impacts of vaccination, prescription-only standards for antimicrobial use, or increasing the frequency of contact between producers and practitioners.

Due to the nature of the data collection, it should be noted that there are some potential biases present in this dataset. There is some potential for over-estimation of the use of antimicrobials for treatment of disease, given that the reporting veterinarian(s) may have recorded antimicrobials used for growth promotion and/or prophylaxis in the treatment field. Furthermore, some diagnostic misclassification may have occurred between diseases that present similarly, as laboratory confirmation was not linked to these records.

This work suggests that failure of antimicrobial treatment is more likely to occur in non-GI conditions, as compared to GI conditions. Furthermore, the use of multiple antimicrobial products is also associated with an increased probability of antimicrobial treatment failure. Improved immunity via vaccination, management and biosecurity strategies may be viewed as a form of antimicrobial stewardship to the industry by reducing required antimicrobial inputs and consequently, reduced selection pressure for AMR. Furthermore, further research is suggested surrounding the economic and health impacts of changes to guidelines surrounding vaccination, antimicrobial acquisition and use, as well as increasing the frequency of contact between producers and practitioners.

## Competing interests

The authors declare that they have no competing interests.

## Authors’ contributions

SGK: carried out analyses and drafted the manuscript. RA and RMF participated in the design of the study, data collection and interpretation of results. DLP was involved in the data analyses, manuscript revision, and interpretation. RRS BA and SAM were involved in the revision of the manuscript and the interpretation of data. All authors approved of the final manuscript.
